# Engineering oncolytic bacteria as precision cancer therapeutics: design principles, therapeutic strategies, and translational perspectives

**DOI:** 10.1093/procel/pwaf085

**Published:** 2025-10-23

**Authors:** Lingxue Niu, Zhenqiang Deng, Yiyu Jin, Ningzi Guan, Haifeng Ye

**Affiliations:** Shanghai Frontiers Science Center of Genome Editing and Cell Therapy, Biomedical Synthetic Biology Research Center, Shanghai Key Laboratory of Regulatory Biology, Institute of Biomedical Sciences and School of Life Sciences, Shanghai Academy of Natural Sciences (SANS), East China Normal University, Shanghai 200241, China; Shanghai Frontiers Science Center of Genome Editing and Cell Therapy, Biomedical Synthetic Biology Research Center, Shanghai Key Laboratory of Regulatory Biology, Institute of Biomedical Sciences and School of Life Sciences, Shanghai Academy of Natural Sciences (SANS), East China Normal University, Shanghai 200241, China; Shanghai Frontiers Science Center of Genome Editing and Cell Therapy, Biomedical Synthetic Biology Research Center, Shanghai Key Laboratory of Regulatory Biology, Institute of Biomedical Sciences and School of Life Sciences, Shanghai Academy of Natural Sciences (SANS), East China Normal University, Shanghai 200241, China; Shanghai Frontiers Science Center of Genome Editing and Cell Therapy, Biomedical Synthetic Biology Research Center, Shanghai Key Laboratory of Regulatory Biology, Institute of Biomedical Sciences and School of Life Sciences, Shanghai Academy of Natural Sciences (SANS), East China Normal University, Shanghai 200241, China; Shanghai SynBioEra Biotechnology Co., Ltd., Shanghai 200241, China; Shanghai Frontiers Science Center of Genome Editing and Cell Therapy, Biomedical Synthetic Biology Research Center, Shanghai Key Laboratory of Regulatory Biology, Institute of Biomedical Sciences and School of Life Sciences, Shanghai Academy of Natural Sciences (SANS), East China Normal University, Shanghai 200241, China; Shanghai SynBioEra Biotechnology Co., Ltd., Shanghai 200241, China

**Keywords:** synthetic biology, precision cancer therapy, oncolytic bacteria, gene circuits

## Abstract

Engineered oncolytic bacteria are emerging as a promising platform for precision cancer therapy, combining inherent tumor tropism, immunogenicity, and programmable gene control. Advances in synthetic biology now enable inducible and autonomous circuits that sense exogenous inputs (chemical signals or physical signals), bacterial self-cues (quorum sensing, bacterial invasion switches, or nitric oxide-responsive promoters), and tumor-specific pathophysiology (hypoxia, low pH, or lactate). These designs regulate colonization, lysis, and the spatiotemporally confined release of therapeutic cargos—including prodrug-converting enzymes, cytokines, and antibody/nanobody fragments—thereby enhancing antitumor efficacy while limiting off-target toxicity. Beyond monotherapy, oncolytic bacteria integrate with complementary modalities—including immune checkpoint blockade, adoptive cell therapies (CAR-T/NK), radiotherapy/chemotherapy, nanomedicine, and oncolytic viruses—to amplify immune activation and to enable multimodal, synergistic regimens. Concurrently, biosensor modules transform bacterial chassis into programmable “microbial factories” that couple therapy with real-time imaging and adaptive responses within the tumor microenvironment. This review synthesizes design principles for bacterial gene regulation, surveys recent preclinical advances, and highlights emerging combination strategies, while outlining translational considerations for safety, manufacturability, dosing, and patient selection. Together, these developments position engineered oncolytic bacteria as a promising route toward safe, effective, and ultimately personalized bacteria-based cancer therapeutics.

## Introduction

Cancer continues to be a leading cause of death worldwide, and its prevalence is steadily rising (Murray, 2024). While conventional cytotoxic therapies can effectively eliminate cancer cells, they often cause severe side effects due to collateral damage to healthy tissues. Thus, achieving precise tumor targeting without harming normal tissues is essential for improving patient outcomes. Recent advances in molecularly targeted therapy and immunotherapy have propelled the field of precision oncology. Molecularly targeted therapies specifically target signaling pathways within tumor cells, allowing for accurate attacks on cancer cells while minimizing damage to healthy tissues (Min and Lee, 2022). Nevertheless, their clinical application is constrained by restricted tumor-type applicability, acquired resistance, and treatment-related toxicities (Mössner, 2022). Immunotherapy harnesses and modulates the host immune system to recognize and eliminate cancer cells by enhancing or reprogramming immune responses. While successful in certain cancers, many tumors remain resistant to immunotherapy due to challenges such as: (i) insufficient immune cell infiltration due to physical barriers in solid tumors, (ii) cytokine release syndrome (CRS) triggered by overactivation, and (iii) off-target effects and limited tumor-homing capability. These challenges highlight the pressing demand for alternative therapeutic approaches to overcome resistance and refine tumor treatment (Emens et al., 2017; Tang et al., 2023; Ye et al., 2023).

In contrast to conventional drugs, which accumulate through passive diffusion, live bacteria can actively penetrate deep into tumors, bypassing aggregation near blood vessels (Dang et al., 2001; Zhang and Forbes, 2015). The unique properties of the tumor microenvironment (TME) allow bacteria to preferentially replicate and colonize tumors. For example, *Salmonella* has been observed to localize to tumors at more than 10,000 times the density found in normal tissues (Yi et al., 2020). Live bacteria offer distinct advantages over traditional anticancer agents by amplifying antitumor effects through inherent tumor-targeting capabilities, potentially enhancing specific immune recognition (Huang et al., 2021b). However, balancing the requirement for bacteria to evade host antimicrobial defenses while stimulating antitumor immunity within the TME remains a challenge. A recent study revealed a unique hysteresis-mediated mechanism in which preexisting interleukin-10 (IL-10) receptor expression across diverse cell types in the TME is exploited by bacteria to protect tumor-associated macrophages (TAMs) from neutrophil phagocytosis, while simultaneously expanding and activating exhausted tumor-resident CD8^+^ T cells (Chang et al., 2025). Several bacterial species, such as *Escherichia*, *Salmonella*, *Listeria*, and *Pseudomonas*, have exhibited the capacity for selective colonization, penetration, and induction of oncolytic tumor regression (Forbes, 2010; Lee et al., 2025). Many studies have demonstrated that oncolytic bacteria can regress tumors effectively, yet challenges such as insufficient therapeutic efficacy, systemic toxicity, and unpredictable bacterial replication complicate their clinical use (Zhou et al., 2023).

Advances in synthetic biology allow the rational design of optimized oncolytic bacterial strains by attenuating virulence factors and integrating customizable therapeutic payloads, with several candidates already progressing into clinical evaluation (Gurbatri et al., 2022; McNerney et al., 2021). Fine-tuning the spatiotemporal control of bacterial therapeutic activity is essential for maximizing drug accumulation, improving resource efficiency, and reducing harm to healthy tissues. To this end, engineered oncolytic bacteria often utilize regulated gene expression systems, incorporating specific promoter elements, to allow for precise control of therapeutic payload delivery *in vivo* (Cubillos-Ruiz et al., 2021; Riglar and Silver, 2018). Classical microbiology typically exploits native microbial traits through strain screening (Xu et al., 2023), adaptive evolution (Crook et al., 2019), and either pathogen attenuation or probiotic optimization (Detmer and Glenting, 2006). By contrast, synthetic biology prioritizes rational and modular design, integrating programmable sensors, genetic circuits, and effectors to deliver precise, tunable, multilayer regulation of bacterial behaviors and therapeutic outputs.

**Figure 1.**
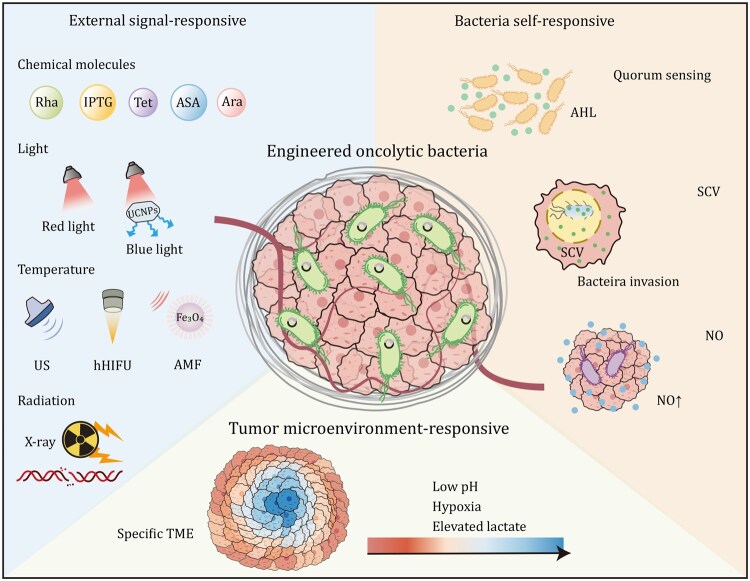
**Controllable gene expression strategies for oncolytic bacteria-mediated precise tumor therapy**. External signal-induced systems utilize chemical inducers (e.g., l-rhamnose (Rha), isopropyl β-d-1-thiogalactopyranoside (IPTG), tetracycline (Tet), acetyl salicylic acid (ASA), and l-arabinose (Ara)), light (red or blue light), temperature modulation via ultrasound (US) or high-intensity focused ultrasound (hHIFU), alternating magnetic field (AMF), and radiation (e.g., X-rays) to trigger gene expression. Bacterial self-responsive systems exploit intrinsic bacterial processes, such as quorum sensing via N-acyl homoserine lactones (AHL) or nitric oxide (NO) production by bacteria as triggers. Additionally, *Salmonella*-containing vacuoles (SCVs) sense the intravacuolar environment after bacterial invasion, activating SPI-2 promoters for tumor-specific payload release. Tumor microenvironment (TME) features, including hypoxia, low pH, elevated lactate levels, and nutrient abundance, also serve as inducers for precise therapeutic gene expression.

This review categorizes and evaluates controllable gene expression strategies for oncolytic bacteria-mediated tumor therapy (Fig. 1). We discuss various genetic control systems, which can be broadly classified into three categories: (i) external signals triggered bacterial gene expression in response to exogenous stimuli, (ii) bacterial self-triggered gene expression which depends on intrinsic bacterial signaling mechanisms, and (iii) pathological signals activated bacterial gene expression in response to tumor-specific microenvironment cues. We also outline both the opportunities and challenges in bridging preclinical findings and clinical applications for engineered oncolytic bacteria and discuss potential future directions for advancing their therapeutic potential.

## Strategies for engineering bacteria

Precisely regulating the expression of therapeutic payloads in oncolytic bacteria has the potential to improve the efficacy of localized tumor therapy while reducing off-target effects (Ozdemir et al., 2018). The rapid advancements in synthetic biology have enabled the design and integration of various gene expression regulatory elements and gene circuits, thus enhancing the precision of engineered bacteria for therapeutic applications (Pedrolli et al., 2019; Teixeira and Fussenegger, 2024). This control is particularly crucial for the expression of potent antitumor effectors, such as cytotoxic compounds and cytokines, whose constitutive production could lead to severe off-target toxicity (Forbes, 2010; Zhou et al., 2018).

## Exogenous input-responsive gene circuits

External cues enable on-demand activation of therapeutic genes only at desired sites or times (Zhou et al., 2018). There are diverse exogenous stimuli, including chemical signals, physical signals (light, temperature, and radiation), to remotely regulate bacterial gene circuits. Such systems provide several advantages: (i) precise temporal control over therapeutic delivery, (ii) reduced metabolic burden during systemic circulation, and (iii) enhanced safety due to dose-titratable activation. Table 1 provides a summary of external signal-responsive bacterial therapeutic systems designed for antitumor applications.

**Table 1.** Exogenous input-responsive gene circuits in oncolytic bacteria for tumor therapy.

Induction signals	Bacterial strain	Therapeutic playload	Administration route	*In vivo* tumor model	References
IPTG	*E. coli* Nissle 1917	LuxCDABE	Intravenous injection (5 × 10^6^ CFU)	Subcutaneous MC26 tumor Subcutaneous human colorectal adenocarcinoma LS174T	Danino et al. (2015)
IPTG	*E. coli* Nissle 1917 Δ*kfiC*	Bacterial capsular polysaccharide (CAP)	Intravenous injection (5 × 10^6^ CFU)	Subcutaneous CT26 tumor, Genetic breast pymt-MMTV	Harimoto et al. (2022)
L-arabinose	*S. typhimurium* Δ*ppGpp*	ClyA	Intravenous injection (3 × 10^7^ CFU)	Orthotopic pancreatic cancer aspc-1 Subcutaneous Capan-2 tumor	Tan et al. (2022)
L-arabinose	*S. typhimurium* Δ*ppGpp*	FlaB	Intravenous injection (1 × 10^7^ CFU)	Subcutaneous MC38 tumor	Zheng et al. (2017)
L-arabinose	*E. coli* Top10	ClyA–OVA–mFc (OMV)	Oral administration (1 × 10^9^ CFU)	Subcutaneous MC38 tumor Subcutaneous B16 tumor Lung metastases B16 tumor	Yue et al. (2022)
L-rhamnose	*E. coli* DH5α	OmpA–mDR18	Intravenous injection (1 × 10^9^ CFU) Intratumoral injection (5 × 10^8^ CFU)	Subcutaneous MC38 tumor Subcutaneous B16F10 tumor	Yang et al. (2025b)
Doxycycline	*S. typhimurium* Δ*ppGpp*	ClyA	Intravenous injection (3 × 10^7^ CFU)	Subcutaneous CT26 tumor Subcutaneous hep3b2.1–7 tumor Lung metastases CT26 tumor	Jiang et al. (2013), Nguyen et al. (2022)
ASA	*S. typhimurium* SL7207	Cytosine deaminase codA	Intraperitoneal injection (1 × 10^6^ CFU)	F1.A11 murine fibrosarcoma tumor	Royo et al. (2007)
NIR light (UCNPs convert to blue light)	*E. coli* Nissle 1917	FlaB	Intravenous injection (1 × 10^6^ CFU)	Subcutaneous H22 tumor Subcutaneous 4T1 tumor Orthotopic 4T1 tumor	Zhu et al. (2023)
NIR light (UCNPs convert to blue light)	*E. coli* MG1655	HlyE	Intravenous injection (1 × 10^7^ CFU)	Subcutaneous CT26 tumor	Tao et al. (2023)
NIR light	*P. aeruginosa* Δ*exoS* Δ*exoT* Δ*vfR*	HlyE	Intratumoral injection (5 × 10^7^ CFU)	Subcutaneous A549 tumor	Fu et al. (2023)
NIR light	*S. enterica* 3934 ΔXIV	ClyA Azurin Anti-PD-L1nb Anti-CTLA-4nb	Intratumoral injection (5 × 10^6^ CFU)	Subcutaneous CT26 tumor Subcutaneous A20 tumor Orthotopic CT26 tumor Orthotopic human breast MCF7 tumor Colorectal PDX model	Qiao et al. (2025)
AMF	*E. coli* BL21 *(*Fe3O4 nanoparticles)	CD47nb	Intravenous injection (1 × 10^8^ CFU)	Orthotopic CT26 tumor	Ma et al. (2023)
Ultrasound	*E. coli* Nissle 1917	αCTLA-4 and αPD-L1	Intravenous injection (1 × 10^8^ CFU)	Subcutaneous A20 tumor	Abedi et al. (2022)
Ultrasound	*S. typhimurium* Δ*ppGpp*	IFN-γ	Intravenous injection (1 × 10^8^ CFU)	Subcutaneous 4T1 tumor Orthotopically transplanted H22 liver tumor	Chen et al. (2022)
Ultrasound	*S. typhimurium* VNP20009	PD-L1 nb and Azurin	Intratumoral injection (5 × 10^5^ CFU) Intravenous injection (5 × 10^5^ CFU)	Subcutaneous B16F10 tumor Subcutaneous CT26 tumor Subcutaneous A20 tumor Subcutaneous H22 tumor	Gao et al. (2024)
Ultrasound	*E. coli* MG1655	miRFP720 and Cytolysin A	Intravenous injection (1 × 10^8^ CFU)	Subcutaneous 4T1 tumor Orthotopic glioblastoma tumor	Du et al. (2025)
hHIFU	*E. coli* MG1655	IFN-γ	Intravenous injection (2.8 × 10^7^ CFU)	Subcutaneous 4T1 tumor	Yang et al. (2024)
X-ray irradiation	*S. typhimurium* VNP20009	TRAIL	Intravenous injection (1 × 10^5^ CFU g^−1^ tumor)	Subcutaneous 4T1 tumor	Ganai et al. (2009)
X-ray irradiation	*E. coli* Nissle 1917	Anti-TREM2 scFv	Oral administration (1 × 10^9^ CFU)	Orthotopic CT26 tumor AOM/DSS-induced colorectal tumor Colorectal PDX model	Wang et al. (2025c)
X-ray irradiation	*S. typhimurium* KST0650	sATF6	Intraperitoneal injection (1 × 10^6^ CFU)	Subcutaneous CT26 tumors	Gao et al. (2020)

### Small molecule-responsive circuits

**Figure 2.**
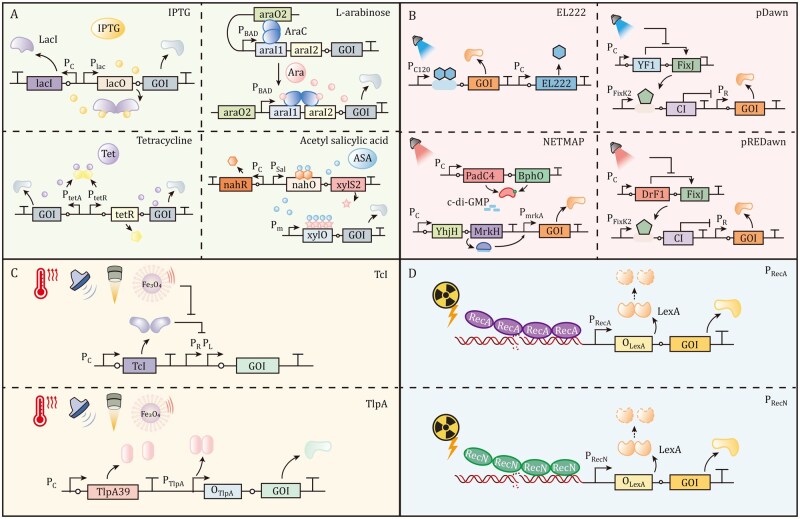
**Bacterial gene expression triggered by external signals**. (A) Chemical inducer-triggered bacterial gene expression. IPTG binds to the LacI repressor tetramer, releasing it from the lacO operator and initiating transcription from the P_lac_ promoter. l-arabinose interacts with the AraC activator, disrupting its binding to araO2 and araI1 sites, thereby allowing RNA polymerase to access the P_BAD_ promoter and initiate transcription. Tetracycline binds to the TetR repressor, causing its dissociation from the tetO operator and enabling transcription initiation from bidirectional promoters P_tetA_ and P_tetR_. Acetyl salicylic acid (ASA) binds to the transcriptional regulator NahR, which induces XylS2 expression from the P_sal_ promoter; XylS2 subsequently promotes transcription from the P_m_ promoter via a synergistic effect. (B) Optogenetic control of gene expression. Blue light-activated EL222 undergoes conformational changes into an oligomeric DNA-binding form, enabling transcriptional activation. In the pDawn system, blue light represses YF1 kinase phosphorylation of FixJ, reducing cI repressor expression and permitting transcription from the P_R_ promoter. The NETMAP system uses 710 nm NIR light to activate PadC4, producing the secondary messenger c-di-GMP, which binds to transcriptional activator MrkH to drive transcription from the P_mrkA_ promoter. In pREDawn, YF1 is replaced by NIR-responsive DrF1, enabling gene regulation under NIR light illumination. (C) Temperature-responsive gene expression. The temperature of the target tissue can be modulated using ultrasound (US), high-intensity focused ultrasound (HIFU), or alternating magnetic field (AMF). In the TcI system, elevated temperatures induce conformational changes that release TcI from its DNA-binding sites, permitting transcription from P_L_ and P_R_ promoters. Similarly, temperature-sensitive repressor TlpA loses DNA-binding capacity upon exposure to hyperthermia, activating downstream genes. (D) Radiation-inducible gene expression. DNA damage resulting from X-ray irradiation triggers RecA or RecN complexes with single-stranded DNA, stimulating autoproteolysis of the LexA repressor. LexA degradation derepresses gene transcription from P_recA_ or P_recN_ promoters.

The control of bacterial gene expression using chemical inducers typically relies on the operon model, where a repressor protein binds to specific DNA sequence to regulate bacterial gene expression in response to added chemical molecules (Fig. 2A) (English et al., 2021; Jacob and Monod, 1961). Chemical inducers interact with regulator proteins or structural enzymes, and allosterically modulate their activity. Chemical inducers such as isopropyl β-d-1-thiogalactopyranoside (IPTG) (Chen et al., 2024; Harimoto et al., 2022; Ma et al., 2023), l-arabinose (Tan et al., 2022; Wei et al., 2016, 2024; Yue et al., 2022; Zheng et al., 2017), rhamnose (Yang et al., 2025b), tetracycline (Jiang et al., 2013; Nguyen et al., 2022, 2024), and salicylic acid salts (Royo et al., 2007), have been employed to control bacterial transgene expression for antitumor applications. These chemical triggers exhibit several favorable features: (i) effective gene regulation at relatively low concentrations; (ii) high bioavailability to penetrate bacterial cells; (iii) stability over the required treatment duration; and (iv) in many cases, relative safety for human administration.

The IPTG-triggered gene expression system is one of the most widely used for high-yield recombinant protein production and for generating antitumor payloads (Marbach and Bettenbrock, 2012). In this system, IPTG binds to the LacI repressor tetramer, releasing it from the *lacO* operator and initiating transcription from the P_lac_ promoter. For example, *Escherichia coli* Nissle 1917 (EcN) expressing lacZ under IPTG control (termed PROP-Z) enabled non-invasive detection of liver metastasis via urinary lacZ signals following oral administration (Danino et al., 2015). Additionally, IPTG inducer has been used to dynamically regulate capsular polysaccharide expression on bacterial surfaces. This transient encapsulation allows bacteria to temporarily evade host immune attack, whereas subsequent capsule loss facilitates effective clearance and enhances the maximum tolerated dose of bacterial therapy by 10-fold, thereby enhancing the therapeutic safety and effectiveness of engineered oncolytic bacteria (Harimoto et al., 2022).

However, despite its widespread use in laboratory studies, the IPTG system suffers from inherent limitations for *in vivo* applications, including challenges in efficient inducer delivery, basal leakiness of gene expression, increased metabolic burden on the host, and potential cellular toxicity (Lutz and Bujard, 1997). As an alternative, the l-arabinose-inducible system offers tighter regulation with minimal leaky expression and a high induction ratio (Guzman et al., 1995). Without l-arabinose, the AraC dimer associates with two distant DNA sites, generating a characteristic DNA loop that inhibits RNA polymerase access. The binding of l-arabinose induces a conformational shift in AraC, stimulating transcription from the P_BAD_ promoter (Englesberg et al., 1969). This system has been successfully utilized to control therapeutic gene expression in oncolytic bacteria. For instance, *E. coli* engineered with the l-arabinose-inducible system expressed cytolysin A, which directly lyses cancer cells (Tan et al., 2022), and flagellin B, an excellent adjuvant for antitumor immunotherapy (Zheng et al., 2017). Moreover, tumor antigen ovalbumin fused to an Fc fragment was expressed in outer membrane vesicles (OMVs) of engineered *E. coli* upon oral co-administration with l-arabinose to function as a potent oral tumor vaccine in mice (Yue et al., 2022). However, the l-arabinose-inducible system exhibits moderate expression efficiency due to relatively low maximum expression levels (Balzer et al., 2013). Recently, the l-rhamnose-inducible system has been employed to express DR18 on the outer membrane in *E. coli* DH5α, which elicited strong CD8^+^ T-cell- and natural killer (NK) cell-dependent antitumor immunity (Yang et al., 2025b). Since l-arabinose and l-rhamnose are natural metabolic substrates, maintaining tight inducibility requires bacterial strains with inactivated arabinose/rhamnose catabolism genes and constitutive expression of arabinose/rhamnose transporters (Liang et al., 2015). These limitations of these systems constrain their broader application in therapeutic settings.

An ideal inducible system should impose minimal metabolic burden and exhibit negligible toxicity. Thus, FDA-approved inducers are highly desirable. Tetracycline and its analogue doxycycline, both antimicrobial agents, have been repurposed for bacterial gene expression regulation (Williams et al., 2010). In the tetracycline-inducible system, the TetR repressor binds to the *tetO* operator sequence, preventing transcription from the bidirectional promoters P_tetA_ and P_tetR_. For example, *Salmonella typhimurium* Δ*ppGpp* engineered with a tetracycline/doxycycline expression system expressed cytolysin A (ClyA) under the P_tetA_ promoter and Renilla luciferase (Rluc8) under the P_tetR_ promoter. Both gene expressions were doxycycline-dependent and significantly suppressed tumor growth in mice (Jiang et al., 2013; Nguyen et al., 2022). However, reliance on antibiotics in such systems is problematic, as it may disrupt bacterial viability and contribute to concerns about antibiotic resistance in clinical settings (Wilson et al., 2020). This highlights the need for safe, widely approved small-molecule inducers without such risks (Cubillos-Ruiz et al., 2021). Acetyl salicylic acid (ASA), a widely used analgesic and anti-inflammatory drug with rapid absorption, broad tissue distribution, a short half-life, and low toxicity, represents a promising alternative (Fuster and Sweeny, 2011). The ASA-inducible system involves two regulators: the naphthalene degradation pathway regulator NahR and the xylose operon transcriptional activator mutant XylS2. In the presence of ASA, NahR activates transcription from the P_sal_ promoter to produce XylS2. ASA simultaneously activates XylS2, which subsequently drives robust expression from the P_m_ promoter via synergistic regulation (Cebolla et al., 2002). In a study, the ASA-regulated *S. typhimurium* SL7207 expresses cytosine deaminase, enabling the conversion of 5-fluorocytosine into the chemotherapeutic agent 5-fluorouracil, led to significantly smaller tumor volumes in mice compared to those regulated by the tetracycline system (Royo et al., 2007).

Despite these advances, small molecule-driven gene expression systems still face several limitations, including cytotoxicity, off-target effects, resistance development, metabolic disturbances, complex pharmacodynamics, and low bioavailability, hampering their broad clinical application (Chait et al., 2016; Kong et al., 2017). Promoter engineering strategy can be introduced to optimize chemical-induced systems. The characteristics of a promoter are largely dictated by its cis-regulatory components, such as the −10 and −35 elements (Wittkopp and Kalay, 2011). Conventional genetic engineering methods, like saturation mutagenesis or error-prone PCR, are often laborious and time-consuming (Gilman and Love, 2016). New approaches, including conditional toxin expression-based screening (Małachowska and Olszewski, 2018) and AI-assisted frameworks like DeepSEED (Zhang et al., 2023), facilitate the efficient selection of promoter variants with desired regulatory properties. Moreover, the challenge of eliminating residual chemical inducers and the poor tissue penetration of small molecules complicate the spatial and temporal precision of gene expression control.

### Light-responsive circuits

Optogenetics enables precise regulation of transcription through the combination of light-sensitive proteins (e.g., photoreceptors from plants or microbial opsins) with regulatory elements (Bansal et al., 2023). Light serves as an effective tool for precision medicine due to its non-invasiveness, spatiotemporal specificity, reversibility, and remote operation (Ye and Fussenegger, 2019). Initially applied to modulate neuronal electrical activity (Bi et al., 2006), optogenetic tools have been adapted for a wide range of biomedical applications, including metabolic disease and tumor therapy, highlighting their potential in disease treatment (Zhou et al., 2025). The integration of optogenetic tools into engineered bacteria for precise regulation of bacterial functions presents a promising approach for tumor therapy. We classify bacterial optogenetic tools according to their sensitivity to specific light wavelengths (Fig. 2B).

Blue light-induced optogenetic systems, depending on the light-oxygen-voltage (LOV) domain (Crosson et al., 2003), such as light-activated transcription factor EL222 (Zoltowski et al., 2013) and pDawn (Ohlendorf et al., 2012), have been widely utilized in bacterial tumor therapy. The EL222-based system, derived from *Erythrobacter litoralis* HTCC2594, consists of an N-terminal LOV domain and a C-terminal NarL/LuxR-type helix–turn–helix (HTH) DNA-binding domain. In the dark, EL222 exists as an inactive monomer stabilized by inhibitory LOV–HTH contacts. Under blue light stimulation, EL222 converts into an oligomeric DNA-binding form, enabling photoactivation of gene transcription (Zoltowski et al., 2013). The pDawn system employs a light-responsive kinase (YF1) and its cognate response regulator (FixJ) to drive expression of the λ phage repressor cI from the P_FixK2_ promoter, which represses transcription from the strong λ promoter P_R_. Illumination with blue light reduces YF1 kinase activity and suppresses cI expression, thereby relieving P_R_ repression and inducing target gene expression (Ohlendorf et al., 2012). While EL222 offers a more compact design with a single light-responsive component, the pDawn system achieves higher induction efficiency due to its phosphorelay cascade, amplifying the light-triggered signal.

However, all blue light-induced systems suffer from limitations such as restricted tissue penetration and potential phototoxicity, restricting their *in vivo* applications. To address these issues, lanthanide-doped upconversion nanoparticles (UCNPs) have been employed (Lamon et al., 2024), which absorb near-infrared (NIR) light and convert it into blue light *in vivo*, enabling deep-tissue optogenetic activation with reduced phototoxicity. Zhu et al. (2023) constructed UCNP-conjugated EcN (EcN-FlaB-UCNPs) expressing flagellin B mediated by EL222, which, under 808 nm irradiation, triggered immune responses such as TAM repolarization and cytotoxic T-cell infiltration, resulting in significant tumor regression in multiple subcutaneous tumor and metastatic tumor models with negligible side effects. Similarly, Tao et al. (2023) engineered *E. coli* MG1655 with the Dawn system conjugated to UCNPs, demonstrating improved tumor suppression with NIR light illumination through HlyE perforin expression and secretion. However, repeated NIR light irradiation at high intensity (0.6 W.cm^−2^) poses a risk of thermal tissue damage, and the need for continuous modification of bacteria with UCNPs raises concerns about potential toxicity when used *in vivo* (Gnach et al., 2015).

Red light-responsive optogenetic systems, including Cph1/OmpR (Schmidl et al., 2014), iLight (Kaberniuk et al., 2021), pREDusk (Multamäki et al., 2022), OptoCre-REDMAP (Jafarbeglou and Dunlop, 2024), and RfpABC (Sun et al., 2025), have been designed to control bacterial gene expression. However, the *in vivo* applicability of these systems remains limited due to low transcriptional activation and the need for continuous illumination. For instance, Fu et al. engineered an attenuated *Pseudomonas aeruginosa* strain with an NIR light-responsive diguanylate cyclase (BphS), which catalyzes the synthesis of the bacterial secondary messenger c-di-GMP and programmable bacterial lifestyle. NIR illumination triggers bacterial lysis and antitumor toxin HlyE release, resulting in tumor regression in A549 tumor mouse models (Fu et al., 2023). The NIR light-inducible NETMAP system, based on a chimeric bacteriophytochrome (PadC4), also demonstrated robust gene activation (up to 55-fold) (Qiao et al., 2025). PadC4 synthesizes c-di-GMP upon 710 nm NIR illumination, which activates the c-di-GMP-responsive activator MrkH to drive expression of multiple antitumor proteins via the P_mrkA_ promoter in NETMAP-engineered *Salmonella enteritidis* 3934 ΔXIV cells, leading to tumor growth inhibition in multiple mouse models by inducing adaptive immune responses and promoting tumor cell apoptosis and lysis.

However, these systems depend on c-di-GMP, a bacterial secondary messenger that governs key processes including motility, cell cycle progression, biofilm formation, and virulence (Jenal et al., 2017). This reliance increases the risk of interference with host endogenous pathways, potentially disrupting bacterial physiology. An alternative approach involves orthogonal red light-inducible systems. Sun et al. developed a far-red light sensor in *E. coli* based on the knotless phytochrome RfpA and its cognate response regulators RfpC and RfpB (collectively termed RfpABC), achieving a maximum dynamic range exceeding 230-fold (Sun et al., 2025). Moreover, small red-light-sensitive proteins have been engineered as versatile optogenetic tools by selecting their specific binding partners through phage display (Le et al., 2025). When coupled with split enzymes (e.g., split-Cre or split-T7 RNA polymerase), these tools provide a pro missing platform for developing next-generation red-light-inducible gene expression systems in bacteria.

### Temperature-responsive circuits

Although red light offers better tissue penetrability than blue light, its penetration depth is still limited to a few millimeters, thereby restricting the clinical application of light-based gene regulation. Consequently, developing engineered bacteria controllable by exogenous signals with enhanced tissue penetration represents a critical research direction.

Ultrasound and magnetic fields are increasingly explored in biomedical engineering owing to their non-invasiveness, spatiotemporal precision, safety, and deep tissue penetration (Maresca et al., 2018; Sitti and Wiersma, 2020). Ultrasound- or magnetic-induced gene expression systems typically exploit localized temperature increases by converting mechanical energy into heat, which can then trigger bacterial gene expression through temperature-sensitive genetic elements (Huang et al., 2021a; Liang et al., 2015). Various temperature-responsive systems have been engineered to control gene expression in bacteria, including the lambda phage-derived thermally inducible P_L_/P_R_ promoters, temperature-sensitive variants of the phage lambda cI protein, and the thermolabile repressed protein TlpA (Fig. 2C). By selecting appropriate ultrasound or magnetic parameters, the target tissue can be kept within an optimal temperature range to initiate gene expression, enabling noninvasive and spatiotemporally controlled bacterial activation in deep tissues.

A temperature-induced system based on thermally inducible promoters P_L_/P_R_ has been developed, which is regulated by the temperature-sensitive repressor TcI42 (Lewis et al., 2011). At 37°C, the TcI42 repressor constitutively prevents the expression of serine integrase Bxb1, which mediates DNA sequence inversion. Upon thermal stimulation at 42°C, relief of TcI42-mediated repression induces Bxb1 expression, which inverts the constitutive P_7_ promoter and strongly drives the expression of therapeutic payloads. Ultrasound-triggered release of immune checkpoint inhibitors (CTLA-4 and PD-L1) markedly suppressed tumor growth by activating *in situ* immune responses (Abedi et al., 2022). In another study, the gene encoding interferon-gamma (IFN-γ) was inserted downstream of the P_L_/P_R_ promoters and regulated by temperature-sensitive mutant cI857 repressor in *E. coli* MG1655. Following intratumoral delivery, focused ultrasound was applied to raise the local temperature to 45°C, triggering IFN-γ expression and activating potent antitumor immunity in subcutaneous and orthotopic liver tumor models (Chen et al., 2022). However, heating tissues to 42–45°C can result in thermal injury to healthy tissues. Ideally, these systems should respond to lower temperatures (39–40°C) with higher controllability and sensitivity. Gao et al. (2024) constructed a sono-activatable integrated gene circuit (SINGER) system utilizing the temperature-sensitive repressor TlpA_39_, which responds to hyperthermia at 39°C. Upon ultrasound irradiation, SINGER produced therapeutic cargos (azurin or PD-L1 nanobody) that suppressed tumor progression and significantly extended survival in multiple mouse tumor models.

Ultrasound-related techniques, including positron emission tomography (PET) and magnetic resonance imaging (MRI), can be integrated with native or engineered bacteria to improve *in situ* visualization of tumors and bacterial localization (Kang and Min, 2021). When coupled with synthetic genetic circuits and biosensors, engineered bacteria can report on tumor presence, burden, and even microenvironmental conditions. Du et al. engineered *E. coli* MG1655 with the TcI repressor and P_L_/P_R_ promoter to construct a real-time imaging-guided ultrasound activatable tumor-targeted therapy mode. Upon ultrasound-mediated activation, the expression of therapeutic molecule cytolysin and the optical imaging probe miRFP720 were enhanced. The fluorescence signal from miRFP720 enabled non-invasive monitoring of therapeutic expression levels *in vivo*. This imaging signal-guided approach allowed precise modulation of gene expression, maintaining therapeutic protein production at optimal levels and demonstrating efficacy (Du et al., 2025). Additionally, Yang et al. engineered *E. coli* MG1655 to biosynthesize gas vesicles (GVs), enabling real-time imaging to guide high-intensity focused ultrasound (hHIFU)-triggered IFN-γ expression, while further conjugating the bacteria with doxorubicin (DOX). In this design, IFN-γ activates cytotoxic immune cells, repolarizes macrophages from an immunosuppressive M2 phenotype to a proinflammatory M1 phenotype, and promotes dendritic cell (DC) maturation, while DOX is released within the acidic TME to induce immunogenic cell death. The synergistic action of IFN-γ and DOX stimulates tumor-specific T-cell responses, resulting in enhanced antitumor efficacy (Yang et al., 2024).

Fe_3_O_4_ nanoparticles are often used in conjunction with an alternating magnetic field (AMF) to convert magnetic energy into heat, which triggers engineered temperature-sensitive bacteria to express therapeutic proteins. For instance, *E. coli* BL21 was engineered with P_L_/P_R_ promoters to drive expression of bacterial lysis proteins. Upon exposure to AMF signals, the Fe_3_O_4_ nanoparticles generate heat that induces the expression of lysis proteins, causing bacterial lysis and release of the pre-expressed anti-CD47 nanobody. This strategy enables spatially controlled release of immunotherapeutic drugs within the TME, minimizing systemic toxicity and offering precision tumor immunotherapy (Ma et al., 2023). However, this approach requires exogenous nanoparticle modification, which may raise safety concerns regarding off-target heating or potential toxicity to healthy tissues. Magnetotactic bacteria, inherently containing magnetic materials, are capable of sensing and responding to external magnetic fields without the need for synthetic nanoparticles. A magnetotactic bacteria-based platform has been developed for the mechanical modulation and suppression of tumor cells. Specifically, RGD (Arg-Gly-Asp) peptide-modified magnetotactic bacteria target and bind to tumor cell surfaces, inducing sustained magnetomechanical oscillations that trigger Ca^2+^ influx *in vitro* and inhibit tumor progression *in vivo* (Wang et al., 2022).

Recently, advanced thermal delivery techniques such as hHIFU and external beam radiation have been employed to achieve refined spatial heat control and to synergize with other therapeutic modalities. However, these approaches often require coupling agents and specialized equipment, increasing the complexity of clinical implementation.

### Radiation-responsive circuits

Radiation possesses strong tissue-penetrating capabilities and can activate gene promoters responsive to DNA damage, enabling spatial and temporal control of gene expression while minimizing systemic toxicity (Noda et al., 2023). Radiation-induced systems in bacteria typically rely on the P_RecA_ or P_RecN_ promoters, which are integral components of the bacterial SOS DNA repair system that ensures genomic stability (Anderson and Kowalczykowski, 1998). Under non-activating conditions, the LexA repressor binds to a specific operator sequence (called SOS box), preventing transcription. When DNA damage occurs, such as through X-ray irradiation, RecA or RecN forms a complex with single-stranded DNA, triggering the autoproteolysis of the repressor and initiating transcription (Fig. 2D). To improve the efficiency of radiation-inducible systems, a CheO box was incorporated into the P_RecA_ promoter region, significantly increasing mTNF-α secretion—by approximately 10-fold—following irradiation in *Clostridium* (Nuyts et al., 2001a). Ganai et al. engineered *S. typhimurium* to express murine TNF-related apoptosis-inducing ligand (TRAIL) under the control of P_RecA_ promoter. Following 2 Gy γ-irradiation, the engineered *S. typhimurium* significantly delayed growth of mammary tumors and reduced tumor-related death by 76% in murine models (Ganai et al., 2009). Additionally, EcN was engineered with a radiation-inducible P_RecA_ promoter to release anti-TREM2 scFv, which precisely targets TREM2-expressing TAMs via OMVs. Under 8 Gy X-ray irradiation, orally administered engineered EcN significantly improved therapeutic efficacy in both orthotopic low rectal cancer and AOM/DSS-induced colorectal cancer models (Wang et al., 2025c).

The P_RecN_ promoter, with lower basal activity compared to the P_RecA_ promoter, is particularly suitable for tumor therapy applications, as its minimal leakage reduces the risk of off-target toxicity (Nuyts et al., 2001b). An attenuated *S. typhimurium* KST0649 strain was genetically modified to express spliced activating transcription factor 6 (sATF6) under the radiation-inducible P_RecN_ promoter, enabling dose-dependent expression of therapeutic proteins in response to irradiation. This strategy led to complete suppression of tumor growth and survival protection in murine tumor models (Gao et al., 2020). Despite these advances, radiation-inducible systems exhibit basal leakiness. Furthermore, deep-seated tumors require high-energy radiation for adequate tissue penetration, which could increase the risk of damage to surrounding healthy tissues.

## Autonomous bacterial signal-responsive gene circuits

**Figure 3.**
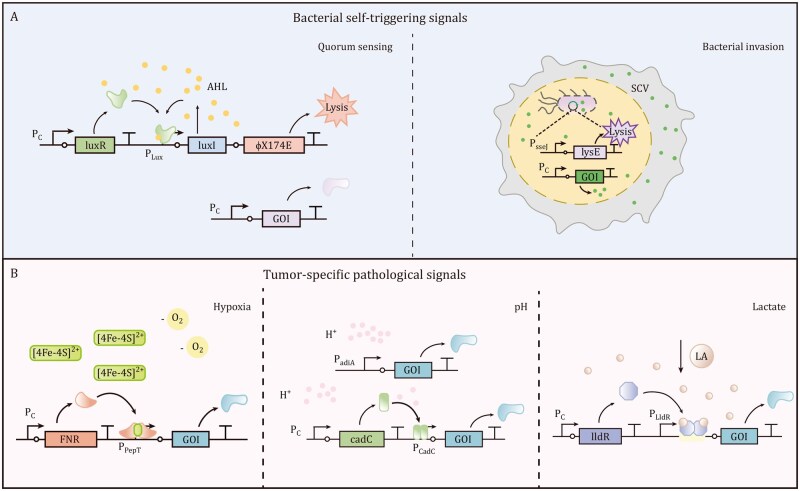
**Internal signal-triggered bacterial gene expression**. (A) Bacterial self-triggering gene expression. Quorum sensing relies on N-acyl homoserine lactones (AHLs) to activate the transcriptional regulator LuxR, which subsequently induces expression of *luxI* (encoding an AHL synthase) and the lysis gene *ϕX174E*, leading to bacterial lysis and release of pre-expressed therapeutic proteins. In *Salmonella, the* formation of Salmonella-containing vacuole (SCV) within host cells allows bacteria to sense specific metabolic cues, such as low pH, which activates *lysE* expression under PsifB promoter, resulting in bacterial lysis and release of constitutively expressed therapeutic agents. (B) Tumor microenvironment (TME)-responsive gene expression. Hypoxia-responsive gene expression is mediated by the transcription factor FNR, which dimerizes via a [4Fe–4S]2+ cluster and binds to palindromic DNA sequences to initiate transcription. Low pH induces gene expression either directly via high concentrations of protons (H+) at the PadiA promoter or through activation of the transcriptional regulator CadC, which initiates gene expression from the PCadC promoter. Lactate-induced gene expression occurs when lactic acid (LA) binds to the LldR dimer, causing its release from the PLldR promoter and thereby enabling transcription of downstream genes.

A distinct class of gene expressed systems repurpose intrinsic bacterial biological processes—such as population communication and host interaction responses—into precise therapeutic triggers. These systems exploit endogenous bacterial signals that are selectively amplified within the TME. Broadly, bacterial self-triggering mechanisms can be classified into three categories: quorum sensing (QS), nitric oxide-responsive activation, and invasion-responsive activation (Fig. 3A). Bacterial self-triggering mechanisms for antitumor effects are summarized and displayed in Table 2.

**Figure 4.**
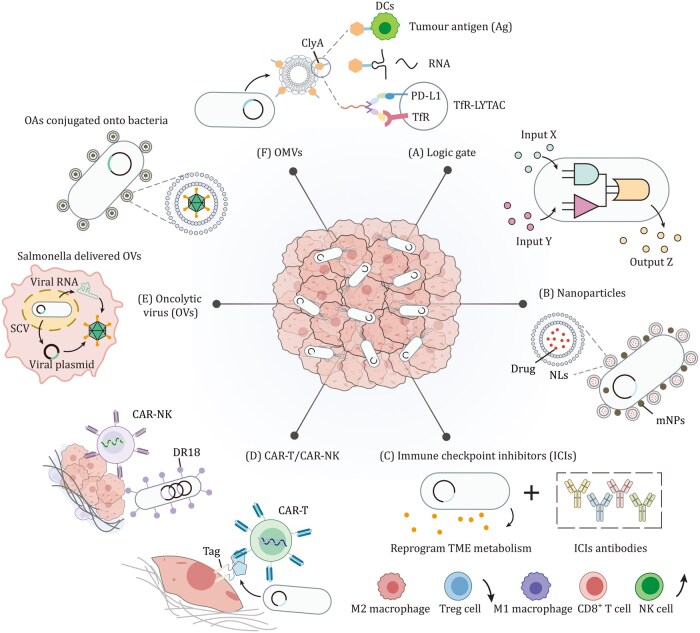
**Future perspectives of engineered bacterial combination therapies**. (**A**) Logic-gated circuits. Engineered bacteria integrate multiple tumor-associated signals through logic gate circuits, producing therapeutic outputs only when specified inputs are simultaneously present. (B) Combination with nanoparticles. Bacteria conjugated with nanoliposomes (NLs) loaded with drug and/or magnetic nanoparticles (mNPs), enabling deep tumor penetration and spatiotemporal control via external magnetic or acoustic fields. (C) Combination with immune checkpoint inhibitors (ICIs). Engineered bacteria can modulate TME metabolism by producing metabolites such as l-arginine and l-tryptophan, enhancing antitumor immune cells (M1 macrophages, CD8+ T cells, and NK cells) while suppressing immunosuppressive populations (M2 macrophages and Treg cells), thereby improving ICI efficacy. (D) Combination with CAR-T or CAR-NK cells. Engineered bacteria can deliver synthetic CAR targets (e.g., GFP-Tags) *in situ* to label tumor cells for recognition and elimination by GFP-specific CAR-T cells. Alternatively, bacteria engineered to display decoy-resistant IL-18 mutein (DR18) enhance immune cell infiltration and prolong CAR-NK cell persistence within the TME. (E) Combination with oncolytic viruses. SCV-based delivery system enables bacteria to transport DNA or RNA encoding oncolytic viruses into tumor cells for *in situ* production of oncolytic viruses. Additionally, oncolytic viruses conjugated to bacterial surfaces via lipid linkers leverage bacterial tumor-homing properties for enhanced tumor targeting. (F) Bacteria outer membrane vesicles (OMVs). Engineered OMVs serve as multifunctional delivery vehicles by presenting tumor antigens (Ag) to enhance dendritic cell uptake, delivering RNA cargos (e.g., mRNA or siRNA) to tumor cells to regulate the expression of specific proteins involved in antitumor responses, and carrying LYTACs to degrade extracellular tumor proteins such as PD-L1.

**Table 2.** Autonomous bacterial signal-responsive gene circuits in oncolytic bacteria for tumor therapy.

Induction signals	Bacterial strain	Therapeutic playload	Administration route	*In vivo* tumor model	References
SLC	*E. coli* NiCo21 (DE3)	CD47nb	Intravenous injection (5 × 10^6^ CFU) Intratumoral injection (1 × 10^7^ CFU)	Subcutaneous A20 tumor Subcutaneous B16F10 tumor Subcutaneous 4T1 tumor	Chowdhury et al. (2019)
SLC	*E. coli* Nissle 1917	PD-L1nb, CTLA-4nb and GM-CSF	Intratumoral injection (1 × 10^7^ CFU)	Subcutaneous A20 tumor Subcutaneous CT26 tumor	Gurbatri et al. (2020)
SLC	*E. coli* Nissle 1917	hCXCL13	Intravesical injection (3 × 10^6^ CFU) Intratumoral injection (1 × 10^6^ CFU)	Orthotopic MB49 tumor Orthotopic UPPL1541	Rouanne et al. (2024)
SLC	*E. coli* Nissle 1917	CXCL16 and CCL20	Intratumoral injection (1 × 10^6^ CFU or 5 × 10^6^ CFU) Intravenous injection (5 × 10^6^ CFU)	Subcutaneous A20 tumor Subcutaneous MC38 tumor Subcutaneous EO771 tumor	Savage et al. (2023)
SLC	*E. coli* Nissle 1917	IFN-γ	Intratumoral injection (2 × 10^6^ CFU) Intravenous injection (2 × 10^7^ CFU)	Subcutaneous MC38 tumor Subcutaneous CT26 tumor	Li et al. (2024b)
SLC	*S. typhimurium* VNP20009	TNFSF14	Gavage administration (8 × 10^8^ CFU)	Transgenic Apc^min/+^ mice AOM/DSS-induced colorectal tumor	Mi et al. (2025)
Bacterial invasion	*S. typhimurium* VNP20009 *ΔflhD ΔsifA ΔsseJ*	Caspase-3	Intratumoral injection (4 × 10^7^ CFU) Intravenous injection (1 × 10^7^ CFU)	Subcutaneous 4T1 tumor Subcutaneous BNL-MEA tumor	Raman et al. (2021)
Bacterial invasion	*S. typhimurium* VNP20009 *ΔflhD Δasd*	OVA	Intratumoral injection (2 × 10^7^ CFU)	KPC pancreatic tumors Subcutaneously MC38 tumor	Raman et al. (2023)
Bacterial invasion	*S. typhimurium* LH1301	SVA virus RNA	Intratumoral injection (2.5 × 10^6^ CFU)	Subcutaneous H446 tumor	Singer et al. (2025)

### Quorum sensing-driven circuits

QS is a bacterial cell–cell communication mechanism that modulates population density-dependent behaviors through the accumulation of diffusible signaling molecules. Once these molecules reach a certain concentration, they interact with regulatory proteins to induce target gene expression through QS promoter (Waters and Bassler, 2005). Since bacteria reach high densities specifically within the TME, QS systems have been exploited to induce tumor-specific expression of therapeutic proteins (Swofford et al., 2015). The most widely used QS system is derived from *V. fischeri* and comprises the transcriptional regulator LuxR, the signaling molecule acyl-homoserine lactones (AHLs) synthase LuxI, and an intergenic luxR-luxI region harboring the P_luxR_ and P_luxI_ promoters (Stevens and Greenberg, 1997).

Dessel et al. emphasized QS as a promising tool for cancer treatment, enabling targeted drug delivery to tumors while minimizing toxicity to normal tissues (Swofford et al., 2015). Subsequently, Tal Danino et al. developed a synchronized lysis circuit (SLC) in which bacteriophage-derived lysis genes are expressed under QS control for tumor therapy. Specifically, the SLC consists of a common P_luxI_ promoter that regulates the production of the autoinducer (AHL), which binds to LuxR and activates transcription (positive feedback), along with a lysis protein (ϕX174 E) also controlled by the P_luxI_ promoter. As AHL diffuses to neighboring cells at a threshold population density, it triggers the release of genetically encoded cargo. After bacterial lysis, a small number of remaining bacteria begin to produce AHL anew, enabling continuous drug delivery within tumors (Din et al., 2016). The SLC has been used to release various therapeutic proteins, including antagonist of CD47 nanobody (CD47nb) (Chowdhury et al., 2019), programmed cell death-ligand 1 (PD-L1) nanobody, cytotoxic T-lymphocyte-associated protein-4 (CTLA-4) nanobody, granulocyte-macrophage colony-stimulating factor (GM-CSF) (Gurbatri et al., 2020, 2024), IFN-γ (Li et al., 2024b), tumor necrosis factor superfamily member 14 (TNFSF14) (Mi et al., 2025), and chemokines such as hCXCL13 (Rouanne et al., 2024), CXCL16, and CCL20 (Savage et al., 2023), facilitating immune cell recruitment and activation and enhancing antitumor immunity.

QS-based systems enable autonomous regulation with spatial and temporal precision, confining bacterial activity to tumors and facilitating continuous therapeutic delivery. However, these systems rely on achieving a threshold bacterial density, which may be difficult to reach in heterogeneous tumor regions, particularly in micro-metastases or invasive fronts. Moreover, the use of bacteriophage-derived lysis genes exerts selective pressure, leading to compensatory mutations that may reduce therapeutic payload release.

### Nitric oxide-responsive circuits

*S. typhimurium* infiltrating tumors has been reported to upregulate nitric oxide synthase (iNOS) expression, causing nitric oxide (NO) levels to increase by as much as 1000-fold, reaching micromolar concentrations within tumor tissue (Barak et al., 2010). Wang et al. designed an NO-inducible system based on the regulatory protein NorR and the P_norV_ promoter, which responds to increased NO. Without NO, NorR interacts with its binding site within the P_norV_ promoter, obstructing RNA polymerase recruitment and repressing transcription. Upon NO binding, NorR undergoes a conformational change, activating transcription from the P_norV_ promoter. This system drives the DNA recombinase FimE expression, which irreversibly inverts the fimS switch to sustain target gene expression. *In vivo* results demonstrated high tumor-to-normal organ ratios for the target gene Rluc8, confirming tumor-specific activation (Qin et al., 2023). These findings indicate that NO serves as a promising inducer for precise spatial control of target gene expression by tumor-targeting bacteria. However, integrating potent therapeutic genes into this NO-responsive genetic circuit is essential for demonstrating antitumor efficacy in rigorous preclinical models.

### Invasion/intracellular niche-responsive circuits

Bacteria can be classified as either intracellular or extracellular based on the parasitism site. Extracellular bacteria reside and proliferate outside host cells, such as *Bacillus anthracis*, *Staphylococcus aureus*, *Pseudomonas aeruginosa*, *Helicobacter pylori*, and *E. coli* (Lynch et al., 2022). In contrast, intracellular bacteria, such as *Fusobacterium*, *Mycobacteria*, *Shigella*, *Brucella*, *Listeria*, *Salmonella*, and *Rickettsia*, inhabit and replicate within host cells (Méresse et al., 1999). A widely studied *Salmonella* utilizes a type 3 secretion system (T3SS)—needle-like complexes spanning the bacterial inner and outer membranes—to deliver effectors into host cells (Kubori et al., 1998). After entering the host cell cytoplasm, *Salmonella* forms the *Salmonella*-containing vacuole (SCV), and the intracellular metabolic signals (e.g., low pH and low phosphate) trigger the expression of the *Salmonella* pathogenicity island 2 (SPI-2) and T3SS-2 genes (Steele-Mortimer, 2008). The T3SS needle complexes then penetrate through the SCV membrane, enabling payload protein secretion into the host cytosol.

The unique invasive properties and intracellular lifecycle of intracellular bacteria present a promising opportunity for engineering novel tumor therapy approaches. Wu et al. developed an efficient SARS-CoV-2 vaccine using *Salmonella* engineered with the SPI-2-specific P_sifB_ promoter. This system enabled high-efficiency delivery of SARS-CoV-2 antigens to antigen-presenting cells (APCs). After co-incubation with macrophages, the P_sifB_ promoter was activated, driving expression of recombinant SARS-CoV-2 antigens, which were delivered via the SPI-2 effector protein sseJ (Wu et al., 2022).

The intrinsic invasion and survival machinery of intracellular bacteria enable the delivery of therapeutic proteins directly into cancer cells. Raman et al. developed an intracellular delivery (ID) system based on the *S. typhimurium* VNP20009, containing an SPI-2-specific genetic circuit (P_sseJ_-lysE). In tumors, *Salmonella* naturally invades cancer cells, where the P_sseJ_ promoter activates lysE expression, resulting in bacterial lysis and the release of constitutively expressed therapeutic proteins (e.g., Casp-3, ovalbumin) into the cytoplasm of infected cells. This targeted delivery approach significantly decreased tumor growth and reduced established metastases in a 4T1 murine breast cancer model (Raman et al., 2021, 2023). Additionally, the ID system-engineered *Salmonella* has been used to deliver plasmid DNA. Khanduja et al. developed a virus-delivering *Salmonella*-based platform to deliver the genome of the oncolytic virus minute virus of mice (MVMp) into cancer cells. Following bacterial invasion and lysis, the plasmids encoding the MVMp genome were released and produced infectious virions, leading to selective oncolysis of the infected tumor cells (Khanduja et al., 2024).

However, the SCV physically confines bacteria and limits the transport of the host cytosol, restricting delivery efficiency. Singe et al. co-expressed hemolysin E (HlyE), a pore-forming toxin, under the P_sseJ_ promoter to disrupt SCV membranes and facilitate vacuolar escape (Nguyen and Portnoy, 2020). Deletion of the *sifA* gene, which destabilizes the SCV membrane, also enhances the delivery efficiency of bacterial cargo (Beuzón et al., 2000; Schroeder et al., 2010). An optimized delivery system has successfully delivered oncolytic Seneca Valley virus RNA into tumor cells, initiating robust oncolytic viral replication, leading to complete regression of subcutaneous small cell lung cancer (SCLC) tumors and achieving 100% survival in treated mice (Singer et al., 2025).

The bacterial T3SS in *Yersinia enterocolitica*, which enables the delivery of therapeutic proteins into eukaryotic tumor cells, has reached phase III clinical trials. However, bacterial invasion lacks inherent specificity, posing a potential limitation (Ittig et al., 2015). *Salmonella* re-expressing flhDC under an arabinose-induced promoter demonstrated specific invasion efficiency in the tumor, improving invasion in 84% of host cells compared to knockout controls (Raman et al., 2021). Another strategy involves surface display of synthetic adhesins on the bacterial outer membrane, such as an epidermal growth factor receptor (EGFR) nanobody, enabling specific binding to tumor cells and improving the selectivity of the T3SS system for protein cargo delivery (Asensio-Calavia et al., 2024). However, most intracellular bacteria are pathogenic species, limiting their clinical application due to inherent virulence (Carlsson and Brown, 2006; Ray et al., 2009). Strategies to mitigate this issue include the construction of auxotrophic strains and the deletion of virulence genes. Alternatively, introducing functional T3SS into nonpathogenic strains represents a promising approach to achieve safe and effective ID (Reeves et al., 2015).

## Tumor microenvironment-responsive gene circuits

Besides external inducers, engineered bacteria can intelligently decode pathological signals within tumors to actuate spatially precise therapeutic gene expression. This approach capitalizes on bacterial innate sensing mechanisms coupled with synthetic gene circuits that transduce tumor-specific microenvironment cues into localized anticancer responses. In contrast to externally triggered gene expression and bacterial self-triggering systems, tumor-specific pathological signal-responsive systems enable regulating bacterial activity within tumors, eliminating the need for complex external control.

Following systemic administration, therapeutic bacteria distribute to the tumor as well as normal tissues. While bacteria in normal tissues are rapidly eliminated within hours and days, those within tumors persist, proliferate, and evade immune responses. Tumor-induced angiogenesis acts as a major contributing factor in this process by facilitating the delivery of oxygen and nutrients. Nevertheless, the abnormal, leaky, and poorly organized structure of tumor blood vessels hinders immune cell infiltration. In addition, hypoxic and inflammatory lesions generate microenvironments that support the survival and expansion of anaerobic and facultative anaerobic bacteria (Clairmont et al., 2000; Min et al., 2008; Quispe-Tintaya et al., 2013). Tumor tissues typically have oxygen concentrations below 1%–2%, markedly lower than 4.6%–9.5% found in normal tissues (Xin et al., 2023). Similarly, the extracellular pH of the TME (6.5–6.9) is consistently lower than that of normal tissues (∼7.3) (Estrella et al., 2013). These unique physicochemical characteristics of the TME can serve as physiological and pathological signals to activate engineered bacterial gene expression in a tumor-specific manner (Fig. 3B). The tumor-specific pathological signals to induce antitumor effects are summarized in Table 3.

**Table 3.** Tumor microenvironment-responsive gene circuits in oncolytic bacteria for tumor therapy.

Induction signals	Bacterial strain	Therapeutic payload	Administration route	*In vivo* tumor model	References
Hypoxia	*E. coli* MG1655	aCD47	Intravenous injection (1 × 10^7^ CFU)	Orthotopic CT26 tumor	Xie et al. (2025)
Hypoxia	*E. coli* Nissle 1917 Δ*dapA* Δ*thyA*	DacA	Intratumoral injection (1 × 10^9^ CFU)	Subcutaneous CT26 tumors Subcutaneous B16F10 tumor	Leventhal et al. (2020)
Hypoxia	*S. typhimurium* SL7207 Δ*asd* Δ*htrA*	ASD	Intravenous injection (1 × 10^7^ CFU)	Subcutaneous MB49 tumors Subcutaneous B16F10 tumor Dextran sodium sulfate (DSS)/azoxymethane (AOM)-induced *in situ* colon tumorsb16f10 lung metastases tumors	Chang et al. (2025)
Hypoxia	*S. typhimurium* SL7207	ASD	Intravenous injection (5 × 10^7^ CFU)	Orthotopic MDA-MB-231 tumor	Yu et al. (2012)
Hypoxia	*S. typhimurium* Δ*htrA* Δ*msbB*	Shiga toxin	Intraperitoneal injection (2 × 10^6^ CFU)	Subcutaneous HeLa tumors	Flentie et al. (2012)
pH	*E. coli* MG1655	ClyA	Intravenous injection (1 × 10^8^ CFU)	Subcutaneous CT26 tumor	Qin et al. (2022)
Lactate and hypoxia	*S. typhimurium* ELH1301 (*Δasd Δglms)*	ASD and GlmS	Intravenous injection (5 × 10^7^ CFU)	Subcutaneous CT26 tumor	Chien et al. (2022)
Lactate	*E. coli* Nissle 1917	Coagulase	Intravenous injection (5 × 10^7^ CFU)	Subcutaneous MC38 tumor	Zou et al. (2025)
Ammonium chloride	*E. coli* Nissle 1917 *ΔargR*	ArgA	Intratumoral injection (5 × 10^6^ CFU) Intravenous injection (5 × 10^7^ CFU)	Subcutaneous MC38 tumor	Canale et al. (2021).

### Hypoxia-responsive circuits

Hypoxia-responsive gene expression systems are based on the fumarate and nitrate reduction (FNR)-like transcriptional regulator, which is naturally present in facultatively anaerobic bacteria such as *Escherichia* and *Salmonella* (Mazoch and Kucera, 2002). Under hypoxic conditions, FNR forms a homodimer by coordinating with [4Fe–4S]^2+^ cluster and binding to palindromic DNA sequences to promote transcription. Under normoxic conditions, the clusters are degraded, leading to dissociation of the FNR dimer into inactive monomers, which suppresses transcription (Crack et al., 2004).

Several hypoxia-responsive promoters, including P_pepT_ (Strauch et al., 1985), P_pfE_, P_ansB_ (Arrach et al., 2008), P_fnrS_ (Durand and Storz, 2010), and P_adhE_ (Chen et al., 2011), are transcriptionally regulated by FNR. To enhance specificity and reduce leaky expression under normoxic conditions, researchers have engineered optimized promoter variants with modified FNR-binding sites (Yang et al., 2025a). These variants, such as HIP-1 (Mengesha et al., 2006) and FF+20 (Ryan et al., 2009), amplify expression in hypoxia while minimizing leaky expression in normoxia. This hypoxia-inducible strategy serves two therapeutic purposes: improving tumor-targeted therapy and enhancing safety.

Hypoxia-responsive P_fdhF_ promoter restricts CD47 antibodies gene expression to hypoxic tumor regions, reducing off-target effects in normal tissues and boosting macrophage antitumor activity (Xie et al., 2025). Leventhal et al. (2020) designed SYNB1891, a living probiotic that produces dacA from *Listeria monocytogenes* to activate STING, triggering type I interferons under hypoxia-responsive P_fnrS_ promoter. Additionally, hypoxia-responsive promoters can drive the expression of essential genes, such as *asd* in *asd*-deleted bacterial strains, to restrict bacterial proliferation within hypoxic tumor zones, thereby minimizing damage to healthy tissues (Chang et al., 2025; Yu et al., 2012).

### Acidity-responsive circuits

Another hallmark of the TME is its acidity, which results from the metabolic reprogramming of cancer cells. Even under normoxic conditions, cancer cells display the Warburg effect—characterized by elevated glucose consumption and lactate generation—leading to acidification of the extracellular space, with pH values typically ranging from 6.5 to 6.9 (Chen et al., 2025; Gerweck and Seetharaman, 1996).

Using a bioluminescent transposon-based reporter trap, Flentie et al. screened *S. typhimurium* for genes controlled by five pH-sensitive promoters: P_adiY_, P_yohJ_, P_STM1787_, P_STM1791_, and P_STM1793_. By coupling the P_STM1787_ promoter to Shiga toxin expression, they demonstrated the feasibility of a bacteria-mediated tumor-specific therapeutic strategy, showing a 90-fold increase in gene expression within 8 h of exposure to acidic tumor conditions (Flentie et al., 2012). Similarly, the P_adiA_ promoter, which responds to acidic conditions, was used to drive cytolysin A (ClyA) expression in the *E. coli* MG1655 strain, demonstrating pH-sensitive regulation of therapeutic protein expression (Qin et al., 2022). Another example involves the membrane-integrated transcriptional activator CadC and its cadBA operon, which show increased activity in acidic medium compared to neutral pH, particularly at pH levels around 5.8 (Tetsch et al., 2011). Chien et al. (2022) transformed the CadC system expressing enhanced green fluorescent protein (EGFP) into *S. typhimurium* ELH1301 and observed an increase in total fluorescence within the acidic core of tumor spheroids.

### Lactate-responsive circuits

Lactate, a byproduct of aerobic glycolysis, dramatically increases in solid tumors and is considered as a signaling molecule regulating cancer cell behavior, tumor–stroma interactions, and immune responses (de la Cruz-López et al., 2019). While normal serum lactate levels range from 1.5 to 3 mmol/L, concentrations within the tumor rise to 10 to 30 mmol/L, and may exceed 50 mmol/L in necrotic tumor cores (Watson and Delgoffe, 2022). Under lactate-deficient conditions, LldR dimers bind to operator sites O1 and O2 within the P_lldPRD_ promoter, forming a tetramer that represses transcription. In the presence of lactate, the LldR dimer at O2 dissociates, while the dimer at O1 functions as a transcriptional activator, initiating gene expression. LldR from *Corynebacterium glutamicum* is preferred for sensor design due to its insensitivity to glucose and anaerobic conditions (Georgi et al., 2008).

Engineered *S. typhimurium* incorporating a hypoxia sensor (P_PepT_ promoter) and a lactate sensor (P_LldR_ promoter and LldR protein) was programmed with an AND gate so that transgene expression occurred only under concurrent hypoxia and elevated lactate (Chien et al., 2022). This dual-input design enhanced tumor specificity while minimizing off-target activation. However, the LldR-based lactate-responsive system is partially repressed by glucose and anaerobic conditions. Zúñiga et al. engineered a hybrid promoter through directed evolution of LldR operators in *E. coli* (named ALPaGA). This optimized promoter eliminated glucose catabolite repression and oxygen-dependent transcriptional regulation, achieving ∼6.2-fold induction under aerobic, glucose-rich conditions and ∼5.3-fold under anaerobic, glucose-rich conditions (Zúñiga et al., 2021). Building upon these foundations, a novel lactate-induced system was developed with specificity for high lactate concentrations (>5 mmol/L) typical of the TME, independent of glucose and oxygen. By regulating the expression of essential genes such as aspartate-semialdehyde dehydrogenase and coagulase, bacterial growth was restricted to tumor tissues, preventing leakage of therapeutic proteins into normal organs (Zou et al., 2025).

Although tumor-specific pathological signals responsive systems are widely used to achieve high-level expression of therapeutic genes, many native promoters exhibit relatively narrow dynamic ranges. This can limit their effectiveness *in vivo*, as inadequate or excessive basal expression of payloads can compromise efficacy and safety. Additionally, the TME exhibits substantial heterogeneity between different tumor samples (Group Young Researchers In Inflammatory et al., 2021). Hypoxic and acid regions within tumors, with dynamic changes in proximity to blood vessels, lead to fluctuations in bacterial sensing signals (Samuel et al., 2023). Moreover, micro-metastases (<2 mm) lack a fully developed TME, presenting challenges for TME-responsive therapeutic strategies.

Matrix metalloproteinases (MMPs), frequently overexpressed in the TME, have emerged as promising tumor-specific molecular cues (Kessenbrock et al., 2010). In a reported approach, several neoantigen peptides were attached to the outer membrane of *S. typhimurium* via an MMP-cleavable linker. Upon tumor infiltration, the engineered *Salmonella* released the neoantigens in response to elevated MMP levels, enabling localized recruitment and activation of lymphocytes, thereby suppressing tumor growth (Hyun et al., 2021). Recently, an engineered EcN-expressing MMP-sensitive interleukin-15 (IL-15) enabled TME-responsive IL-15 delivery. When combined with photothermal therapy, this system amplified the antitumor effect by promoting the recruitment of APCs and T cells, as well as the expansion of T and NK cells (Wang et al., 2025b). However, pre-expression of neoantigens on the bacterial surface before intravenous administration may lead to premature immune activation during systemic circulation. Therefore, it is essential to develop systems that sense tumor-specific macromolecules and connect cellular signaling to the regulation of gene expression in bacteria.

While synthetic receptors can sense TME components (e.g., tumor antigens, cytokines) for cell-based therapies, translating this strategy to bacteria remains challenging. Specifically, the double-membrane envelope structure of Gram-negative bacteria and the thick peptidoglycan layer of Gram-positive bacteria pose physical barriers to effective recognition and interaction with extracellular signals.

Overcoming this limitation requires new strategies to couple tumor extracellular antigen detection with intracellular bacterial signal transduction. Additionally, metabolic reprogramming offers another avenue for designing context-specific bacterial responses, including the dysregulated availability of glucose, lipids, amino acids, and oxygen (Demicco et al., 2024). Canale et al. developed an engineered EcN by deleting the arginine repressor gene (*ArgR*) and integrating ArgAfbr to enhance l-arginine production. When ammonium chloride was used as the sole nitrogen source, the engineered bacteria converted intratumoral ammonia into l-arginine, increased local l-arginine levels, promoted T-cell infiltration, and improved the efficacy of anti-PD-L1 treatment (Canale et al., 2021). The complex metabolism within the TME highlights the need for deeper investigation, which could inform the design of next-generation bacterial gene circuits capable of precise, context-dependent regulation of therapeutic functions.

## Clinical translation of engineered oncolytic bacteria

In 1891, William Coley pioneered the use of live bacteria in cancer therapy by administering *Streptococcus* to treat bone sarcomas. Although this approach induced tumor regressions, it also led to infection-related fatalities in multiple patients (Nauts et al., 1946). The subsequent bacterial cancer therapy involved intravesical Bacillus Calmette-Guérin (BCG), a live-attenuated *M. bovis*, now employed for the treatment of non-muscle-invasive bladder cancer (Lamm et al., 1980). Intravesical BCG became the first bacterial cancer therapy to gain FDA approval in 1990. Various bacterial strains—including human pathogens such as *Salmonella* spp., *Listeria* spp., and *Clostridium* spp., as well as probiotics like *E. coli* Nissle 1917, *Lactobacillus* spp., and *Bifidobacterium* spp.—have been explored for anticancer applications, with *Salmonella* and *Listeria* receiving the most attention for cancer therapy (Forbes et al., 2018).

To address safety concerns associated with wild-type bacteria causing systemic immune responses, various attenuated strains have been engineered to improve safety profiles while retaining therapeutic efficacy, making them more suitable for clinical application (Table 4). For example, VNP20009, derived from *S. typhimurium* ATCC14028, carries deletions of the *purI* and *msbB* genes, leading to a safer profile through genetically attenuated virulence, reduced septic shock potential, and antibiotic susceptibility (Clairmont et al., 2000). The *S. typhimurium* Δ*ppGpp* strain, a *relA* and *SpoT* double mutant, is unable to synthesize the stringent response regulator ppGpp, demonstrating a virulence in extensive murine models (Na et al., 2006). Another attenuated *S. typhimurium* strain, SL7207, contains a deletion in the *aroA* gene, rendering it incapable of synthesizing aromatic amino acids and restricting its proliferation to the nutrient-rich TME (Medina et al., 1999). The *S. typhimurium* A1-R strain, a leucine and arginine auxotroph, was developed through nitrosoguanidine (NTG) mutagenesis and is designed to selectively grow in tumors where these nutrients are abundant (Zhao et al., 2005). The phoP/phoQ two-component regulatory system of *Salmonella* regulates genes associated with virulence and survival within macrophages; deletion of both *phoP* and *phoQ* reduced virulence and enhanced safety (Miller et al., 1989). Additionally, *S. typhimurium* YB1, which lacks the essential *asd* gene, has been engineered to restrict bacterial growth to hypoxic tumor regions by controlling the gene under a hypoxia-inducible promoter (Yu et al., 2012).

**Table 4.** Attenuated strains of *Salmonella* and *Listeria*.

Strain	Name	Mutation	Property of attenuation	References
*Salmonella*	VNP20009	Δ*purI* Δ*msbB*	Increased tumor chemotaxis, lowered systemic toxicity, and sensitivity to various antibiotics	Clairmont et al. (2000)
	Δ*ppGpp*	Δ*relA* Δ*spoT*	Defective in 5′-diphosphate-3′-diphosphate (*ppgpp*) synthesis to reduce transcription of virulence genes and decrease the mean lethal dose	Na et al. (2006)
	SL7207	*hisG*46, Del407 [*aroA*::Tn1 (Tc-s)]	Unable to produce aromatic amino acids, which are only found in the tumor microenvironment	Medina et al. (1999)
	A1-R	Leu/Arg dependent	Unable to produce Leu and Arg amino acids, which are only found in a nutrient-rich tumor microenvironment	Zhao et al. (2005)
	Δ*phoP* Δ*phoQ*	Δ*phoP* Δ*phoQ*	Decreased survival in cultured mouse macrophages	Miller et al. (1989)
	YB1	Hypoxia-conditioned promoter expressed *asd* gene	Essential gene *asd* is engineered so that it is under the control of a hypoxia-conditioned promoter that selectively targets the tumor microenvironment	Yu et al. (2012)
*Listeria*	*L. monocytogenes* Δ*dal* Δ*dat* Δ*actA*	Δ*dal* Δ*dat* Δ*actA*	Regulating D-alanine metabolism limited *in vivo* survival	Wallecha et al. (2009)
	*L. monocytogenes* Δ*prfA*	Δ*prfA*	Reduces intracellular growth and cell-to-cell spread and complementation with prfa-containing plasmid	Freitag et al. (1993)
	*L. monocytogenes* Δ*actA* Δ*inlB*	Δ*actA* and Δ*inlB*	Deficiency in cell-to-cell spread and reduced capacity to infect	Brockstedt et al. (2004)

*Listeria* has been widely explored as a cancer vaccine vector due to its unique intracellular lifecycle. Following invasion, *Listeria* expresses listeriolysin O (LLO), which forms pores in the phagosome, allowing bacterial translocation into the cytosol and promoting major histocompatibility complex class I (MHC-I)-mediated protein presentation (Ikonomidis et al., 1994). To improve safety, attenuated *Listeria* strains with reduced virulence have been developed. For example, deletion of the *dal* and *dat* genes—responsible for d-alanine synthesis—impairs cell wall biosynthesis and intracellular replication (Wallecha et al., 2009). Deletion of the *prfA* gene, the master regulator of *Listeria* virulence, must be complemented with a *prfA*-containing plasmid (Freitag et al., 1993). Additionally, deletion of *actA* and *inlB* attenuates intercellular spread, preventing invasion of non-phagocytic cells and minimizing systemic dissemination (Brockstedt et al., 2004).

Advances in synthetic biology expand the potential of oncolytic bacteria for cancer therapy. Several engineered strains exhibit promising antitumor effects in preclinical models, enabling precise genetic control, payload customization, and enhanced tumor specificity (Sieow et al., 2021). Currently, engineered oncolytic bacterial strains are undergoing clinical trials (Table 5). These engineered bacteria enhance tumor susceptibility by remodeling the TME, either through the presentation of tumor-associated antigens (TAAs) or the delivery of immune modulators, mitigating cytotoxic T-cell exhaustion. For example, a phase I trial of genetically modified *Bifidobacterium longum* expressing interleukin-12 (IL-12) is underway to stimulate local and systemic immune responses (NCT04025307), while oral *S. typhimurium* expressing IL-2 is being tested in patients with metastatic pancreatic cancer (NCT04589234). A first-in-human trial is evaluating a genetically modified *Yersinia enterocolitica* strain (T3P-Y058-739) using T3SS to inject therapeutic proteins directly into tumor cells (NCT05120596), aiming to overcome PD-1-mediated cytotoxic T-cell exhaustion. Engineered *L. monocytogenes* strains, such as Axalimogene filolisbac (NCT02399813), have shown promise in delivering HPV-16 E7 oncoprotein for cervical cancer patients. Additionally, a recent Phase I clinical trial is evaluating the intratumoral injection of *S typhimurium*-expressing l-methioninase, aiming to starve tumors of essential amino acids as a novel strategy (NCT05103345).

**Table 5.** Recent and ongoing clinical trials with engineered tumor-targeting bacteria.

Bacterial strain	Engineered strategy	Tumor type	Sample size	Mode of Delivery	Phase	Sponsor	References
bacTRL-IL-12 *Bifidobacterium longum*	Express IL-12	Advanced and treatment-refractory solid tumors	5	Intravenous injection	Phase I (terminated)	Iqvia Pty Ltd	NCT04025307
NECVAX-NEO1 *Salmonella typhimurium* Ty21a strain	Express AI predicted the most immunogenic patient-specific neoepitopes	Advanced solid tumors	20	Oral administration in combination with PD-1/PD-L1 mAbs	Phase I/II	NEC Bio B.V	NCT06631079
SGN1 *Salmonella typhimurium*	Express L-methioninase	Histologically confirmed advanced and/or metastatic solid tumors	70	Intratumoral injection	Phase I	Guangzhou Sinogen Pharmaceutical Co., Ltd.	NCT05103345
TXSVN *Salmonella* bacteria	Express Survivin	Multiple myeloma	1	Oral vaccination	Phase I	Baylor College of Medicine	NCT03762291
T3P-Y058-739 *Yersinia enterocolitica*	Express undisclosed type I interferons (IFNs) and toll-like receptor (TLR) proteins, which are delivered into tumor cells via bacterial type III secretion	Advanced solid tumors	100	Part A: intratumoral injection Part B: intravenous injection	Phase I/II	T3 Pharmaceuticals AG	NCT05120596
ADXS 11-001 *Listeria monocytogenes*	Express the HPV-associated protein E6	HPV-positive oropharyngeal squamous cell carcinoma	15	Intravenous injection	Phase II	Andrew Sikora	NCT02002182
SYNB1891 *E. coli* Nissle 1917	Produce c-di-GMP, a stimulator of the STING pathway, which plays a key role in eliciting antitumor immune responses by activating antigen-presenting cells (APCs) and presenting tumor antigens	Advanced/metastatic solid tumors or lymphoma	32	Intratumoral injection	Phase I (terminated)	Synlogic	NCT04167137
ADXS-NEO *Listeria monocytogenes*	Express these participant-specific tumor antigens	Advanced or metastatic solid tumors	13	Intravenous injection and combination with pembrolizumab	Phase I (terminated)	Advaxis, Inc.	NCT03265080
ADXS11-001 *Listeria monocytogenes*	Express a truncated fragment of LLO fused to HPV antigens	High risk locally advanced cervical cancer	110	Intravenous infusions	Phase III (terminated)	Advaxis, Inc.	NCT02853604
ADXS11-001 *Listeria monocytogenes*	Express a LLO fused to HPV-16 E7 oncoprotein	Persistent/recurrent, loco-regional or metastatic squamous cell carcinoma of the anorectal canal	36	Intravenous infusions	Phase II	Advaxis, Inc.	NCT02399813
ADXS31-164 *Listeria monocytogenes*	Express the chimeric human HER2/neu fusion protein	HER2-expressing solid tumors	12	Intravenous infusions	Phase I b	Advaxis, Inc.	NCT02386501
APS001F *Bifidobacterium longum*	Express cytosine deaminase (CD)	Advanced and/or metastatic solid tumors	75	intravenous Infusions followed by oral 5-FU	Phase I/II	Anaeropharma Science, Inc.	NCT01562626
VXM01 *Salmonella typhimurium*	Express VEGFR-2 and anti-PD-L1	Progressive glioblastoma	30	Oral administration and combination with avelumab	Phase I/II	Vaximm GmbH	NCT03750071
CRS-207 *Listeria monocytogenes*	Express tumor-associated antigen mesothelin	Pancreatic cancer	61	intravenous Infusions	Phase II	Sidney Kimmel Comprehensive Cancer Center at Johns Hopkins	NCT03190265
Saltikva *Salmonella typhimurium*	Express the human IL-2	Metastatic pancreatic cancer	60	Oral administration	Phase II	Salspera LLC	NCT04589234
ADXS-503 *Listeria monocytogenes*	Elicit potent T-cell responses against 22 tumor antigens commonly found in NSCLC	Metastatic squamous or non-squamous non-small cell lung cancer	24	intravenous Infusions	Phase I/II	Advaxis, Inc.	NCT03847519

Despite significant progress in clinical trials, most engineered oncolytic bacterial therapies remain in early phases, with most advanced trials in Phase II. The delayed clinical translation is largely due to inconsistencies between preclinical findings and clinical outcomes. Factors such as bacterial accumulation, tumor colonization, immune response elicitation, and immune system clearance in different species and individuals vary widely. Nonetheless, the development of new engineered bacterial therapeutics continues, with an increasing number of bacterial-based therapies progressing to clinical trials. Precision-controlled oncolytic bacteria hold the potential to deliver immune modulators in combination with immune checkpoint inhibitors (ICIs), potentially overcoming resistance to ICIs and boosting host antitumor immunity.

## Future perspectives

Engineered live oncolytic bacteria as “microbial factories” hold the potential to continuously produce multiple therapeutic proteins within tumors, enabling them to fight cancer more effectively. The success of this strategy depends on selecting an optimal bacterial chassis, as different strains exhibit distinct characteristics, including tumor-specific colonization capacity, inherent oncolytic mechanisms, immunomodulatory properties, and genetic tractability. To minimize off-target toxicity, attenuated bacterial strains have been developed through preclinical engineering, enabling preferential tumor targeting. Further precision is achieved by integrating genetically encoded circuits that restrict bacterial activation or proliferation to inducible gene circuits. Therapeutic payloads can be customized to meet the specific needs of different tumor contexts: for “hot” tumors, bacteria deliver immunomodulators (e.g., cytokines, tumor antigens) to amplify antitumor immunity; while for “cold” tumors, they express direct-killing effectors (e.g., toxins, apoptosis proteins) to overcome poor immune infiltration.

The integration of reporter genes (e.g., luciferase, fluorescent proteins) allows for non-invasive imaging to track bacterial distribution and therapeutic efficacy in real-time, integrating therapeutic and diagnostic functions in a single platform. This review focuses on inducible gene expression systems for precise tumor therapy and clarifies mechanisms triggered by external signals, bacterial invasion, and tumor-specific pathological signals. We discuss key design considerations, types of inducers, and therapeutic payloads in different inducible gene circuits (Tables 1–3), providing a reference for optimizing bacterial-mediated therapy and advancing its clinical applicability.

Although external signal-induced gene expression offers greater controllability and precise temporal regulation, tumor-specific pathological signal-triggered systems enable intelligent, self-regulating responses to tumor microenvironment (TEM) cues. These systems utilize feedback mechanisms where bacteria sense tumor-specific signals (e.g., hypoxia, acidosis) and dynamically modulate therapeutic output, achieving theranostic control. By designing tumor-specific pathological signal-triggered systems, therapeutic release can be molecularly tethered to tumor-specific signals in both time and space, enabling bacteria to adapt to tumor progression. Logic-gate designs can enhance precision in tumor therapy. For example, *E. coli* Nissle 1917 has been engineered to release hemolysin under TME conditions of hypoxia, low pH, and high lactate levels, implementing an XOR amplifier to target colorectal cancer (Zhou et al., 2024a). Similarly, an AND logic gate was constructed in *E. coli* Nissle 1917, ensuring therapeutic protein release only in the presence of both lactate and AHL, minimizing leakage (Zou et al., 2025). These highlight the potential of logic gate engineering in the development of intelligent bacterial theranostic systems.

Moreover, engineered live oncolytic bacteria combined with other antitumor strategies like nanomaterials, ICIs, and oncolytic virus therapy, can enhance therapeutic coverage, overcome monotherapy limitations, and amplify tumor-killing capacity (Fig. 4). Clinical studies have demonstrated encouraging results in combining immunotherapies and chemotherapeutic agents (Table 5).

### Integration with nanomaterials

The development of intelligent nanobiohybrids is a promising area of research for effective cancer treatment. Nanomaterials can encapsulate bacteria as drug factories, while also integrating nanophotosensitizers or acoustic sensitizers for synergistic therapies. Nanomedicine improves the efficiency of engineered bacteria, and the bacteria, in turn, augment the performance of the nanomedicine (Zhou et al., 2024b). For example, Xie et al. used *cis*-aconitic anhydride to conjugate DOX onto EcN, achieving TME-triggered chemotherapy with DOX levels 6.1-fold higher in tumors than with non-conjugated cells (Shirai and Tsukada, 2020). A biohybrid microrobotic platform composed of engineered motile bacteria, magnetic nanoparticles (mNPs), and pH- and light-responsive nanoliposomes (NLs) for DOX delivery allows spatiotemporally controlled release of therapeutic agents at the target site (Akolpoglu et al., 2022). Additionally, bacteria functionalized with paramagnetic Fe_3_O_4_ nanoparticles (Ma et al., 2023) or BaTiO_3_ nanocubes (Fan et al., 2024) effectively targeted orthotopic colon tumors. These combined approaches demonstrated the promising potential of engineered bacteria with nanomaterials for cancer therapy.

### Combination with immune checkpoint inhibitors

Engineered bacteria represent a promising strategy to augment cancer immunotherapy by remodeling the TME and improving immune responses (Wang et al., 2025a). ICIs are often limited by poor immune cell infiltration and a highly immunosuppressive TME, which is enriched with regulatory cells (e.g., regulatory T cells, myeloid-derived suppressor cells) and inhibitory cytokines (e.g., TGF-β, IL-10). Recently, genetically modified EcN has been engineered to convert TME metabolic byproducts, such as ammonium, into l-arginine, thereby promoting tumor-infiltrating T cells and enhancing the anti-PD-L1 therapy (Canale et al., 2021). Similarly, engineered *Clostridium butyricum* strains expressing tryptophan synthesis genes (*trpEDCBA*) have shown enhanced metabolic activities of CD8^+^ T cells, resulting in synergistic antitumor effects when combined with PD-L1-blocking antibodies (Wang et al., 2024).

Some bacterial species have a natural function for remodeling the TME. For example, the metabolite indole-3-aldehyde (I3A) produced by *Lactobacillus reuteri*, as well as indole-3-propionic acid (IPA) generated collaboratively by *Lactobacillus johnsonii* and *Clostridium sporogenes*, can enhance T-cell function within the TME and counteract the immunosuppressive effects of tumors, improving the efficacy of immune checkpoint blockade therapies (Jia et al., 2024; Phelps et al., 2025). A recent study also demonstrated that exercise-induced gut microbial one-carbon metabolism enhances cytotoxic CD8^+^ T-cell (Tc1)-mediated ICI efficacy in melanoma suppression (Bender et al., 2023). Future research could focus on reprogramming TME metabolic patterns by engineering bacteria to facilitate this strategy.

### Synergy with CAR-T (chimeric antigen receptor T) or CAR-NK (chimeric antigen receptor natural killer) cells

Bacteria-based cross-kingdom combination therapies offer a promising strategy to improve CAR-T efficacy in solid tumors. Solid tumors pose greater challenges for CAR-T therapy due to the limited availability of conserved, tumor-restricted antigens, which increases the risk of off-target toxicity in healthy tissues. By delivering synthetic antigens within the TME, bacteria enable more precise recognition and targeting of solid tumors by CAR-T cells. For instance, *E. coli* Nissle 1917 was engineered to deliver synthetic antigens (green fluorescent protein GFP) to guide CAR-T cells to solid tumors (Vincent et al., 2023). Similarly, *E. coli* DH5α expressing DR18 enhances tumor trafficking and therapeutic efficacy of CAR-NK cells in mesothelioma models, significantly improving tumor control and survival (Yang et al., 2025b).

### Conjunction with oncolytic viruses

Oncolytic viruses, which specifically target, replicate, and destroy cancer cells, have shown promise when combined with bacterial therapies. For example, tumor-homing bacteria can serve as vectors for the delivery of an oncolytic virus (Singer et al., 2025). Another strategy involves combining oncolytic viruses with the surface of engineered bacteria to improve tumor targeting. *E. coli* BL21, due to its capacity to bind oncolytic adenovirus via lipids, can increase oncolytic adenovirus accumulation in non-small cell lung tumors by approximately 170 times compared to intravenous administration of naked oncolytic adenovirus. This combination significantly decreases tumor growth and increases mouse survival (Sun et al., 2022).

### Utilization of bacterial outer membrane vesicles

Bacterial OMVs have emerged as effective nanodrug delivery systems for cancer therapy. OMVs offer unique advantages over live bacteria, including enhanced safety profiles that reduce the risks of infection, while certain native OMVs mediate potent antitumor effects (Li et al., 2024a). OMVs have been studied as vaccines for tumor antigen (Ag) delivery. For instance, engineered *E. coli* expressing a tumor antigen (ClyA-Ag-mFc) in OMVs were internalized by DCs in the gut, followed by lymph node drainage and tumor antigen presentation, modulating immune responses (Yue et al., 2022). OMVs can also serve as RNA delivery systems, providing stability and sustained delivery for various RNA types, including mRNA, miRNA, and siRNA. For example, Li et al. engineered OMVs as a platform for mRNA delivery (OMV-LL-mRNA) by decorating their surface with the RNA-binding protein L7Ae, which specifically adsorbs box C/D sequence-labelled mRNA antigens. These OMVs, carrying a lysosomal escape protein LLO, delivered the mRNA into DCs, significantly inhibiting melanoma progression and causing 37.5% complete regression in a colon cancer model (Li et al., 2022). Similarly, *E. coli* Nissle 1917 Δ*nlpI* was used to deliver PD-L1 siRNA for tumor treatment, effectively downregulating PD-L1 gene expression by approximately 2-fold and achieving 49.37% tumor suppression in a 4T1 tumor model (Sun et al., 2024). Su et al. developed genetically engineered bacterial OMVs to deliver transferrin receptor lysosome targeting chimera (TfR-LYTAC) to induce lysosomal degradation of extracellular PD-L1 in tumor cells, resulting in 60% complete tumor regression and survival beyond 90 days in CT26 colon carcinoma-bearing mice (Su et al., 2024).

## Conclusion

Advances in synthetic biology are equipping bacterial therapeutics with heightened sensitivity and the capacity to process orthogonal inputs, establishing the foundation for truly precision oncology. Engineered bacteria can be programmed to preferentially accumulate in tumors, remodel the immune microenvironment, and release therapeutic agents with tight spatial and temporal control to improve therapeutic efficacy while minimizing off-target effects. These features position oncolytic bacteria as responsive, modular “living medicines” with an improved safety profile. At the same time, the complex relationship between the microbiome and cancer warrants careful consideration: while certain microorganisms can promote tumor initiation or metastatic spread (Ayabe and White, 2024; El Tekle and Garrett, 2023), elucidating these mechanisms will inform chassis selection, circuit design, and patient stratification. Looking ahead, translational success will hinge on robust biocontainment (auxotrophy, kill-switches, genetic firewalls), manufacturability and dosing standardization, mitigation of horizontal gene transfer, and rational combinations with immunotherapies and conventional modalities. With these elements in place, engineered bacteria are poised to deliver safer, more effective, and personalized cancer treatments.

## Data Availability

No data were used for the research described in the article.
